# The Power of Three in Cannabis Shotgun Proteomics: Proteases, Databases and Search Engines

**DOI:** 10.3390/proteomes8020013

**Published:** 2020-06-15

**Authors:** Delphine Vincent, Keith Savin, Simone Rochfort, German Spangenberg

**Affiliations:** Agriculture Victoria Research, AgriBio, Centre for AgriBioscience, Bundoora, Victoria 3083, Australia; keith.savin@agriculture.vic.gov.au (K.S.); simone.rochfort@agriculture.vic.gov.au (S.R.); german.spangenberg@agriculture.vic.gov.au (G.S.)

**Keywords:** cannabis sativa, bottom-up and middle-down proteomics, post-translational modification, missed cleavages, SEQUEST, Mascot, LC-MS, Asp-N, chymotrypsin, trypsin/Lys-C

## Abstract

Cannabis research has taken off since the relaxation of legislation, yet proteomics is still lagging. In 2019, we published three proteomics methods aimed at optimizing protein extraction, protein digestion for bottom-up and middle-down proteomics, as well as the analysis of intact proteins for top-down proteomics. The database of *Cannabis sativa* proteins used in these studies was retrieved from UniProt, the reference repositories for proteins, which is incomplete and therefore underrepresents the genetic diversity of this non-model species. In this fourth study, we remedy this shortcoming by searching larger databases from various sources. We also compare two search engines, the oldest, SEQUEST, and the most popular, Mascot. This shotgun proteomics experiment also utilizes the power of parallel digestions with orthogonal proteases of increasing selectivity, namely chymotrypsin, trypsin/Lys-C and Asp-N. Our results show that the larger the database the greater the list of accessions identified but the longer the duration of the search. Using orthogonal proteases and different search algorithms increases the total number of proteins identified, most of them common despite differing proteases and algorithms, but many of them unique as well.

## 1. Introduction

Bottom-up proteomics (BUP) refers to the characterization of proteins by analysis of their peptides released through proteolysis. When BUP is performed on a mixture of proteins it is called shotgun proteomics [1,2,3,4]. Large-scale or high-throughput analyses of highly complex samples are commonly accomplished using a BUP strategy [5]. Middle-down proteomics (MDP) [6] also refers to the characterization of proteins through proteolysis, albeit via either purposeful and partial digestion and/or choosing a protease targeting fewer excision sites and therefore yielding longer peptides. As MDP can also be applied to a complex mixture of proteins, it falls under the shotgun proteomics category. In other words, shotgun proteomics is a peptide-centric approach, as opposed to top-down proteomics (TDP) [7], which is a protein-centric approach. In a typical shotgun proteomics experiment, proteins are extracted and digested using one or several proteases, and the peptide mixture is separated using liquid chromatography (LC) once or multiple times and subjected to tandem mass spectrometry (MS/MS) analysis [8]. Using complex search algorithms, the tandem mass spectra derived from peptide fragmentations are compared with theoretical tandem mass spectra generated from in silico digestion of a protein database, and the amino acid (AA) sequences of the peptides are thus obtained. Peptide sequences are assigned to proteins by protein inference and post-translational modifications (PTMs) of identified proteins inferred [9]. 

A key step in performing a successful shotgun proteomics experiment relies on the choice of proteases to convert the proteins present in a sample into a complex mixture of peptides. Proteolytic enzymes differ by their selectivity for cleaving the amide bonds between individual residues in a protein. The cleavage is carried out through hydrolysis of the amide bond before or after one or several specific residues. Trypsin has become the gold standard for protein digestion for shotgun proteomics. Trypsin is a serine protease which cleaves at the carboxyl side of positively charged arginine (R) and lysine (K) residues. Alternative proteases display different selectivity, either being more specific than trypsin by targeting only one AA residue or less specific than trypsin by targeting more than two AA residues. When opting for multiple proteases, it is advisable to choose enzymes that act orthogonally to each other. This is the case for Asp-N, more selective than trypsin, which cleaves at the amine side of the negatively charged residue aspartic acid (D), as well as for chymotrypsin, less selective than trypsin, which cleaves at the carboxyl side of hydrophobic residues phenylalanine (F), tyrosine (Y), and tryptophan (W). The value of adopting a parallel digestion strategy based on orthogonal proteases has been amply demonstrated on various species and research goals [10,11,12,13,14,15,16,17,18]. The outcomes of generating complementary peptides resulted not only in an increased number of identified proteins but also in greater sequence coverage and, consequently, more PTMs being detected [8,19,20,21]. 

Following MS/MS data acquisition of the digests, another critical step in the shotgun proteomics workflow is a bioinformatics component and consists of a database search. First, a protein sequence database must be retrieved; second, a search algorithm must process the database and match it against the experimental data. Protein databases in the form of FASTA AA sequences can be freely downloaded from various sources depending on the species of interest. The two main protein sequence repositories are 1) the UniProt Knowledge Base (https://www.uniprot.org/help/uniprotkb), which is the central hub for the collection of functional information on proteins, with accurate, consistent and rich annotation, and as such is the main reference database for proteins, and 2) NCBI (https://www.ncbi.nlm.nih.gov/protein/), which is a collection of sequences from several sources. Both repositories supply annotated sequences, particularly UniProtKB, which also indicate biological ontologies, pathways, processing, PTMs, isoforms, classifications and cross-references. This information is extremely valuable to the prospective scientist because it gives a quick, yet thorough, snapshot of the knowledge associated with the proteins. 

Data interpretation of the fragmentation spectra used to be performed manually by an expert, which was labour-intensive, time-consuming and of low throughput. MS-based proteomics greatly progressed with the automation of identification of candidate peptide sequences from MS/MS spectra using search algorithms and scoring models which assess the likelihood of a match. In order to model matches to sequences, four basic concepts have been devised: descriptive (e.g., Sequest), interpretative (e.g., PeptideSearch), stochastic (e.g., SCOPE) and probability-based modeling (e.g., Mascot) [22]. SEQUEST was the first search algorithm developed [23]. It leveraged two timely technological developments: 1) the sequencing of genomes and 2) the creation of algorithms to match peptide tandem spectra to peptide sequences. The most popular search engine is Mascot; it relies on the probabilistic matching of fragment ions [24]. Nowadays a multitude of search engines and bioinformatics tools exist [25,26,27,28,29]. An analysis will benefit from the use of multiple search algorithms with improved overall identification numbers and confidence [8,22]. Bioinformatic software packages are now bundled into proteomics pipelines, such as ProteomeDiscoverer (www.thermoscientific.com) [8]. This automation has greatly contributed to help standardize and simplify MS-based proteomic analysis. 

The relaxing of the legislation around cannabis, in particular in a therapeutic context, in the 21st century has triggered a surge in research with more than 22,000 articles published since 1840 and hosted at the National Center for Biotechnology Information (NCBI, https://www.ncbi.nlm.nih.gov/pubmed/?term=cannabis+sativa). It peaked in 2019 with 2120 publications and is already exceeding 1000 articles this year (2020). *Cannabis sativa* was named the “plant of the thousand and one molecules” by Andre and colleagues [30] due to its immense catalog of unique chemical compounds. Recent reviews on the topic are available [31,32,33,34]. Among the therapeutically promising molecules are the phytocannabinoids and terpenes. The nuclear and chloroplastic genomes of *C. sativa* have been sequenced [35,36,37,38] and are predicted to accommodate 27,819 to 34,589 genes [33]. NCBI hosts more than 30,000 *C. sativa* protein entries but UniProt, the reference protein database, hosts less than 500 *C. sativa* accessions. Another source of sequences for this non-model plant species rests within the Medicinal Plant Genomics Resource (MPGR, http://medicinalplantgenomics.msu.edu/) from a genome sequencing collaborative effort. Unfortunately, none of these gene models are annotated in MPGR. Despite the sequencing of the *C. sativa* genome, cannabis proteomics remains underrepresented with only 27 publications since 2004 (including 15 articles since 2018, https://www.ncbi.nlm.nih.gov/pubmed/?term=cannabis+sativa+AND+proteom* cannabis sativa AND proteom*). Legislative hurdles aside, perhaps the lack of *C. sativa* sequence in the reference protein database UniProt has impeded proteomics progress on cannabis research.

This experiment is the fourth installment of a series of proof-of-concept methodological developments aiming at designing screening procedures to rapidly analyse the proteome of mature buds from various cultivars of medicinal cannabis. In a first step, we optimized the extraction of cannabis proteins, demonstrating the superiority of guanidine-hydrochloride over urea [1]. In a second step, we optimized the digestion of cannabis proteins using a multiprotease (four enzymes) and multidigestion (single, double and triple digestion) approach, questioning the rationale behind limiting the number of missed cleavages allowed [2]. In a third step, we developed a top-down proteomics strategy to analyse intact proteins and discovered that cannabis proteins are predominantly methylated [3]. These three proof-of-concept proteomics studies share one deficiency: the UniProt database searched for the purpose of protein identification was small (containing less than 500 entries) and consequently greatly underestimated the rich genetic diversity of *C. sativa* [33]. To remedy the database shortcomings of the three past studies, this fourth experiment endeavors to test four *C. sativa* databases of various sizes, from a database reduced to a metabolic pathway (phytocannabinoids and terpenoids) to a database combining entries from several independent sources and therefore exhibiting some level of redundancy. One of the *C. sativa* databases originates from the proteogenomics study by Jenkins and Osburn [39]. We also search a fifth non-specific database, SwissProt *viridiplantae*, to evaluate what is gained or lost for non-model plant species such as *C. sativa*. We take this opportunity to compare two very popular search engines: SEQUEST and Mascot. Finally, in this fourth experiment, we present shotgun proteomics results related to single parallel digestions using orthogonal proteases of decreasing selectivity, namely Asp-N, Trypsin/Lys-C and chymotrypsin. Our observations are presented and discussed with respect to LC-MS patterns, database search yield and duration, search algorithm comparison, protease efficiency, number of accessions, number of missed cleavages and peptide size, PTMs and gene ontology.

## 2. Materials and Methods 

**Figure 1:**
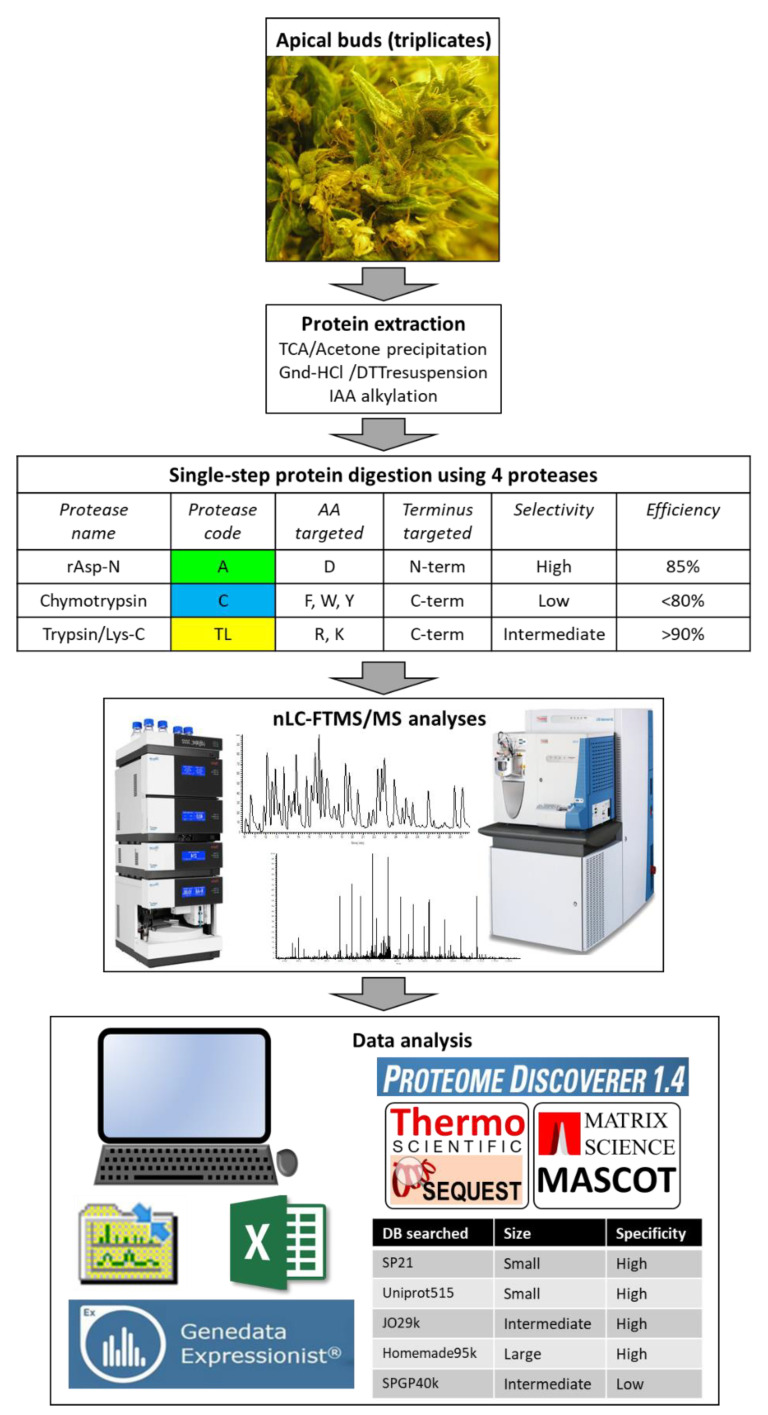
Experimental design.

Figure 1 schematises the experimental design of this study.

### 2.1. Protein Extraction, Digestion, and Analysis Using Nano Liquid Chromatography-Tandem Mass Spectrometry (nLC-MS/MS)

Individual mature apical buds of medicinal cannabis were sampled in triplicate (labelled “bud1”, “bud2” and “bud3” hereafter) and proteins were extracted using a trichloroacetic acid/acetone precipitation followed by resuspension in a guanidine-HCl buffer as detailed in [1,2]. A plant protein content of 100 µg was digested using either a trypsin/Lys-C protease mixture (TL, Mass Spectrometry Grade, 100 μg, Promega), or chymotrypsin (C, Sequencing Grade, 25 μg, Promega) or rAsp-N (A, Sequencing Grade, 10 μg, Promega). Digestions with trypsin/Lys-C and chymotrypsin have been described in [2]. For the digestion using rAsp-N, 50 mM Ammonium bicarbonate (pH 7.8) was added to the dithiothreitol (DTT)-reduced and iodoacetamide (IAA)-alkylated proteins in order to drop the guanidine-HCl resuspension buffer molarity below 1 M. The protease was carefully solubilised in 0.1 mL of ddH_2_O. To achieve a 1:50 ratio of protease:proteins, as instructed by the manufacturer, a 20 µL aliquot of rAsp-N solution was added and gently mixed with the protein extracts. The mixture was incubated at 37 °C in the dark for 1 h. All digests were cleaned up using solid phase extraction (SPE) cartridges followed by evaporation, as explained in [1,2]. Peptides digests were analysed using nLC-MS/MS exactly as described in [1,2].

### 2.2. Protein Identification Using Five Databases and Statistical Analyses

The RAW files were processed in Proteome Discoverer (PD) version 1.4 (PD 1.4, ThermoFisher Scientific) using both SEQUEST and Mascot search engines. The workstation hosting PD 1.4 has a 64-bit operating system, a physical memory of 16 GB RAM, and an Intel® Core™ i7-2600 CPU @ 3.4 GHz processor with 4 cores. PD 1.4 can access our in-house MASCOT server version 2.6.1 which comprises 40 logical and 2 physical Intel processors with 20 cores and 4 CPU units. Five FASTA databases of increasing entry size and two levels of specificity were searched, as listed in Table 1.

SP21, Uniprot515 and Homemade95k databases include two *C. sativa* AA sequences unavailable from public databases and retrieved from patents. The first one is the aromatic prenyltransferase_geranyl-pyrophosphate olivetolic acid geranyltransferase or GOT or CBGAS for short (patent WO/2011/017798A1 or PCT/CA2O1 O/OO1222 [40]). The second one is cannabichromenic acid synthase or CBCAS for short (patent WO/2015/196275Al or PCT/CA20 15/000423 [41]). The SPGP40k database contains all green plant sequences from SwissProt *viridiplantae*, including 19 sequences from *C. sativa*. The JO29k FASTA file is linked to the recent proteomic study of Jenkins and Osburn [39] and contains 29,057 AA sequences, many of which are duplicates. This proved unparsable using the Mascot algorithm and generated many warnings in the SEQUEST search engine. For the Homemade95k database, a collection of 95,069 *C. sativa* protein sequences, in FASTA format, was constructed with 1133 sequences from the UniProt Knowledgebase (with SwissProt and Trembl annotations), 36,525 entries from the GenBank nr protein database (with nr annotations) and 57,411 proteins from the Medicinal Plant Genomic Resource (MPGR). All MPGR sequences were annotated by aligning them to sequences in the GenBank nr database using blastp [42]. Each database was indexed in PD 1.4 using each of the enzymes, which generated the corresponding reversed databases for the decoy searches.

The searching parameters in PD 1.4 specified trypsin, chymotrypsin or AspN as the proteases and allowed for maximum number of missed cleavages, as discussed in [2]. The mass tolerances were set at 10 ppm for the precursor and 0.8 Da for the fragments. Both SEQUEST (Thermo Fisher Scientific Australia Pty Ltd, Scoresby, VIC, Australia) and Mascot (Matrix Science Ltd, London, UK) algorithms were interrogated in parallel. All PD 1.4 parameters are indicated in Supplementary Materials Figure S1. The steps labelled “MASCOT” and “SEQUEST” correspond to the algorithm target searches during which the various databases indicated in Table 1 are searched. Decoy searches are performed during these steps using the reversed decoy database. We exemplify this in Supplementary Materials File F1.txt using the Homemade95k database, which lists step by step the full process of the PD 1.4 workflow along with the durations. The last step of the workflow consists of a target/decoy peptide-spectrum match (PSM) validation which compares the results from both target and decoy databases and eliminates the false positives using a strict false discovery rate (FDR) threshold of 1%.

The nine RAW files are publicly available from the stable public repository MassIVE (http://massive.ucsd.edu/ProteoSAFe/datasets.jsp, reference number MSV000085379). 

The data files obtained following nLC-MS/MS analysis were processed in the Refiner MS module of Genedata Expressionist® 12.0, and statistical analyses were performed using the Analyst module, as detailed in [2]. For each peptide ion, the set of isotopic peaks is grouped into a cluster. The volumes under each peak are summed to make up the volume of the peptide cluster. Cluster volumes were used for the statistical analyses.

## 3. Results and Discussion

The purpose of the present study on medicinal cannabis buds is two-fold: (1) testing several proteases of varying selectivity and efficiency and (2) assessing the relevance of several databases of varying size and specificity as well as comparing two widely used search engines (Figure 1). 

### 3.1. Comparison of the nLC-MS Files

Three digestions were performed on three biological replicates, yielding nine samples. In order to enable meaningful comparisons across proteases, every sample preparation step was kept rigorously identical for each sample (protein extraction, reduction and alkylation, protein amount digested, dilution factor, SPE desalting and LC-MS analyses), with the exception of the digestion steps themselves where optimum conditions were applied as recommended by the manufacturer to maximise protease efficiency. In particular, unique protease:protein ratios (1:50 for A, 1:100 for C and 1:25 for TL), tailored digestion buffers (50 mM Tris pH 8.0 for TL, 100 mM Tris/10 mM CaCl_2_ pH 8.0 for C and 50 mM ammonium bicarbonate pH 7.8 for A), different temperatures (37 °C for A and TL and 25 °C for C) and two digestion times were employed (1 h for A, and 18 h for C and TL). Alternative digestion parameters might lead to improved results and the reader is encouraged to test them; this is however outside the scope of this work.

**Figure 2:**
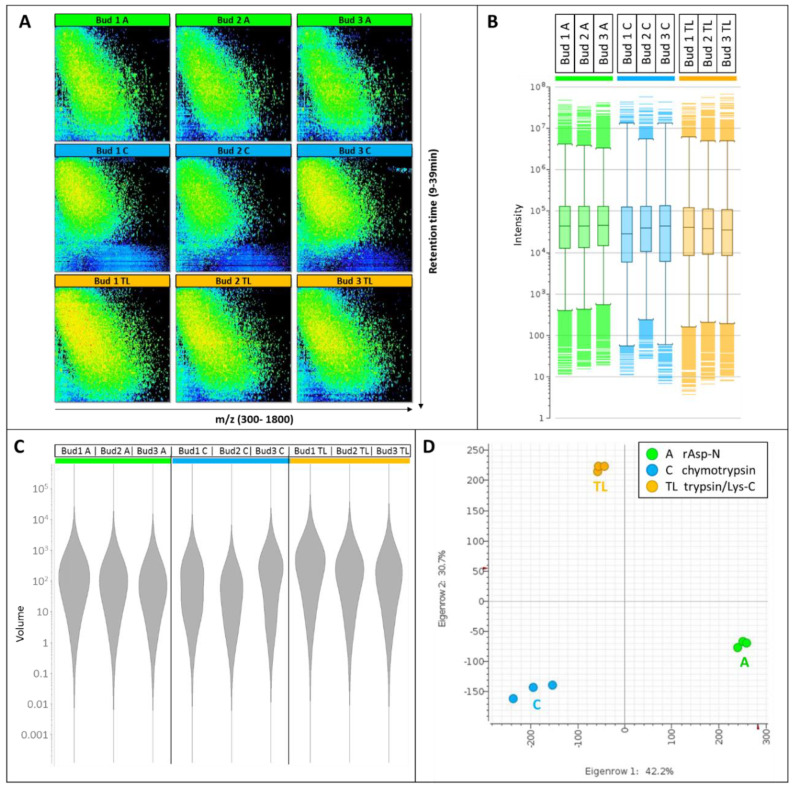
LC-MS pattern and statistical results. (**A**) 2-D nLC-MS maps along m/z 300–1800 on the X-axis and 9–39 min retention time on the Y-axis. (**B**) Box plots of cluster intensities. (**C**) Violin plots of cluster volumes. (**D**) Principal component analysis (PCA) plots of PC1xPC2 of the nine samples. Buds 1–3 are the biological triplicates. Proteases A, rAsp-N; protease C, chymotrypsin; protease TL, trypsin/Lys-C.

All nine nLC-MS maps show reproducible diagonal separation patterns (Figure 2A) demonstrating that low m/z features eluting early are less hydrophobic whereas features characterized by higher m/z elute are late and are therefore more hydrophobic. 

Digest patterns resulting from the action of rAsp-N or trypsin/Lys-C occupy most of the retention time and m/z windows, whereas chymotryptic patterns do not exploit such large windows with only a few peptides greater than m/z 1200 and eluting after 35 min. The depletion in late-eluting hydrophobic peptides released by chymotrypsin is also very evident on the base peak chromatograms (BPC, Supplementary Materials Figure S2). The elution window of the most abundant peptides is protease-specific; prominent peptides elute from 20–30 min when rAsp-N is used, from 15–27 min when chymotrypsin is used and from 17–30 min when trypsin/Lys-C is employed (Supplementary Materials Figure S2). 

The signal intensities are comparable across all samples and stretch from 4 to close to 10^8^ (Figure 2B). A violin plot of the cluster volumes further indicates that the bulk of the volumes range from 10 to 1000 (Figure 2C), with tryptic/Lys-C digests displaying slightly greater volumes. A principal component analysis (PCA) plot demonstrates the high levels of reproducibility of the biological triplicates tightly grouped together, albeit to a lesser extent when chymotrypsin is employed (Figure 2D). The fact that the three digest profiles form a triangular shape on the PCA plot also illustrates how distinct and complementary they are from each other, a testament to their orthogonality. 

The numbers of MS and MS/MS scans per sample are listed in Table 2, along with the number of clusters.

The numbers of MS scans range from 10,391 (bud2_C) to 13,423 (bud1_TL), the numbers of MS/MS scans fluctuate from 8458 (bud2_C) to 11,828 (bud1_TL), and 82,091 (bud2_C) to 91,784 (bud1_A) clusters can be resolved under our nLC-MS conditions. Those numbers are comparable to what was previously reported [1,2], unsurprisingly given that the protein extraction and analytical methods are exactly the same. 

Based on the averages indicated in Table 2, the three proteases rank as follows: TL > A > C. Standard deviations (SDs) are low and coefficients of variation (CV) inferior to 7%. This ranking coincides with the protease:protein ratios (1:25 for TL > 1:50 for A > 1:100 for C); therefore, it would be interesting to repeat this experiment by keeping a consistent protease:protein ratio (for instance 1:100, which is often used in shotgun proteomics) and verifying whether it evens out all the differences highlighted above.

### 3.2. Database Search Yield and Duration

Protein sequences are the fundamental determinants of biological structure and function. A protein sequence database is required to match an acquired spectrum to its theoretical counterpart. The database comprises the AA sequences of all proteins that are expected in the sample. This is why specific protein databases arising from genome sequencing projects of the species of interest are ideal. However, if that is not readily available, sequences from a closely related species must be explored. Issues related to the database search include variant proteins, sequencing errors or homologous proteins from another species [43]. Several *C. sativa* genomes have been sequenced [35,36,37,38], and the number of predicted gene models varies from 27,819 to 34,589 [33]. 

In our previous experiments, a small *C. sativa* FASTA database retrieved from UniProt Knowledge Base was searched to identify proteins from medicinal cannabis apical buds [1,2,3]. This database corresponded to what we refer to as Uniprot515 in the present study. Whilst highly specific, extremely well annotated and originating from a high reputable source, such a small database does not capture the genetic richness of this very unique plant and clearly underrepresents the actual number of *C. sativa* proteins. 

To overcome this shortfall, we have retrieved two significantly larger *C. sativa* FASTA databases from various sources, which we named homemade95k and JO29k (Table 1). We have also searched the non-specific but curated SwissProt *viridiplantae* (green plants) database (SPGP40k), which comprises only 19 *C. sativa* accessions out of close to 40 thousand entries, to test what was gained or lost when the search space was not limited to the species of interest. 

A continued focus of ours are the phytocannabinoid and terpenoid pathways; 21 of the enzymes involved in these metabolisms and reviewed in UniProt (i.e., originating from SwissProt) are gathered in a minimalist FASTA database called SP21. In all, we searched five databases of varying sizes and specificity (Table 1). 

All the proteins identified using the five databases and the two algorithms (SEQUEST and Mascot) for the nine samples are listed in Supplementary Materials Tables S1–S5. When searching the SP21 database, 18 accessions out of 21 (85.7%) are identified across all nine samples. A search with Uniprot515 database produces 72 accessions out of 515 (14.0%) sequences. Exploring the JO29k database yields 1343 accessions out of 29,057 (4.6%). Using the largest database, Homemade95k, 1442 accessions out of 95,069 (1.5%) are found across all nine samples. Finally, interrogating the less specific SPGP40k database leads to the identification of 819 accessions out of 39,800 (2.1%) entries. Identification results are summarized in Table 3.

The number of identities varies from 9 (SP21 Mascot bud123_A) to 1322 (Homemade95k SEQUEST bud1_TL) (Table 3). Within a given database, that number fluctuates by up to 58% (from 549 to 1322 in Homemade95k) across the nine samples. Of course, if we focus our attention on one database, one search engine and one digestion, the number of accessions identified is much more comparable (CVs < 11.5%), further confirming the acceptable reproducibility noted above across the biological triplicates.

There is a clear positive relationship between the number of proteins identified and the size of the database. Considering SEQUEST results only since Mascot could not be applied to JO29k, on average 15 proteins are identified using the SP21 database (72.5% of all the database entries), Uniprot515 produces 54 identifications (13.2%), 1006 accessions are listed with JO29k (3.5%), Homemade95K yields 944 identities (1.2%) and 653 proteins are identified using SPGP40k (1.6%). 

The percentages listed in Table 3 correspond to the number of identities relative to the total number of entries in the database searched; those values show that the greater the database the smaller the percentage. For instance, up to 76% of the accessions listed in the smallest database, SP21, are matched (SEQUEST bud1_TL), but as little as 0.6% of the entries comprised in the largest database, Homemade95k, are identified (Mascot bud3_C). These opposite trends between number of identities and percentages per database are better visualised in the histogram in Supplementary Materials Figure S3A. 

The UniProt Knowledge Base (https://www.uniprot.org/) collates data from SwissProt and TrEMBL, thus providing annotated sets of protein sequences, predicted from sequenced genomes for many species, in particular model organisms. Manually curated and reviewed protein sequences emanate from SwissProt, whereas automatically annotated and unreviewed proteins originate from TrEMBL. Searching against SwissProt thus ensures that the identifications are based on high quality protein information [44]. Limiting the search space to the set of sequences expected in the sample by restricting the database to the species of interest increases the biological relevance of the results. A specific taxonomy can be selected from the UniProt website which covers model organisms better than non-model species such as *C. sativa*, where species-unique proteins are missed. In such cases a related species with similar sequences is to be used; alternatively, if no close relatives exist in UniProt, a whole taxum can be searched. For less studied plant species, the *viridiplantae* taxa is the best taxonomy offered by UniProt. In the case of *C. sativa*, there are currently 19 reviewed (SwissProt) entries and 494 unreviewed (TrEMBL) entries hosted in the UniProt repository. In this work, 72 accessions from the UniProt repository are identified overall, including 17 accessions from SwissProt (Supplementary Materials Table S2).

The NCBI protein database (https://www.ncbi.nlm.nih.gov/protein) gathers sequences from several sources (GenBank, RefSeq and Third-Party Annotation (TPA), SwissProt, PIR, PRF and PDB) and makes them publicly available. GenPept translations exist for each of the coding sequences within the GenBank Nucleotide database; consequently, more than one protein sequence might correspond to a nucleotide sequence record. When UniProt builds become available, they are loaded into NCBI. The RefSeq project at the NCBI (http://www.ncbi.nlm.nih.gov/refseq/) has several missions: maintaining and curating annotated genomic, transcript and protein sequence records; leveraging data submitted to the International Nucleotide Sequence Database Collaboration (INSDC) against a combination of computation, manual curation and collaboration to produce a standard set of stable, non-redundant (nr) reference sequences; adding references to publications, functional features and informative nomenclature [45]. GenBank is a public repository of DNA sequences built from community data submissions to INSDC, as well as daily data exchanges from the DNA DataBank of Japan (DDBJ), the European Nucleotide Archive (ENA) and GenBank at NCBI [46]. We retrieved from the NCBI repository 37,654 *C. sativa* AA sequences that are fully annotated, including 36,521 accessions from RefSeq, 899 entries from TrEMBL and 234 sequences from SwissProt. Overall, 834 accessions originating from the NCBI database are identified in this work (Supplementary Materials Table S4), one from SwissProt and all the others from RefSeq. 

The Medicinal Plant Genomics Resource (MPGR, http://medicinalplantgenomics.msu.edu/) stems from the Medicinal Plant Consortium (MPC). Initiated in 2010 and funded by the National Institutes of Health (NIH), MPC gathers 13 collaborating research units from 7 institutions. MPC aims to provide publicly available transcriptomic and metabolomic resources for 14 key medicinal plants for the worldwide research community for the advancement of drug production and development. It wishes to bridge the gap between genomic information and the highly specialized secondary metabolisms of plants with promising medical applications such as *C. sativa*. A total of 57,411 *C. sativa* AA sequences are available from MPGR, which exceeds the genetic richness mentioned above and therefore might host redundant sequences. MPGR accessions lack protein descriptions, which we added by applying the blastp sequence alignment algorithm [42] to the GenBank nr database. Overall, 608 accessions originating from the MPGR database are identified in this work (Supplementary Materials Table S4). Most of them (530, 87%) match a *C. sativa* protein, 22 (4%) match accessions from *Trema orientale*, and 17 (3%) match proteins from *Parasponia andersonii*. Both species belong to the *Cannabaceae* family and are closely related to *C. sativa* [47]. 

The JO29k database created by Jenkins and Osburn [39] is also publicly available and contains 29,057 entries. The authors employed trypsin to digest protein extracts from various tissues from various cultivars of *C. sativa* plants followed by a fractionation method prior to shotgun LC-MS/MS analyses. By maximizing sample diversity (genetic backgrounds, vegetative and reproductive tissues) and by prefractionating the tryptic digests, they managed to not only achieve extensive proteome coverage with the identification of 17,269 open reading frames but also validate genome annotations using proteogenomics. The authors do not indicate how many proteins were identified in mature female flowers. In our study, using the JO29k database, we identified 1343 accessions in mature buds. 

While it is obviously advantageous to search larger specific databases since they generate longer lists of identifications, it is computationally taxing, particularly when dynamic modifications are added and an unlimited number of miscleavages is allowed, as was done in this study, because all those parameters greatly increase the search space. Table 4 details the search durations for each sample.

For the databases containing less than a thousand entries, search durations take minutes, whereas hours are needed when several thousands of entries are interrogated. For the smallest databases, SP21 and Uniprot515, search durations span from 8 to 12 min and 16 to 26 min, respectively (Table 4). For databases of comparable size, such as JO29k and SPGP40k, search durations fluctuate from 19 min to 1 h 22 min and from 2 h 18 min to 4 h 17 min, respectively. The largest database, Homemade95k, necessitates the longest search durations, from 5 h 21 min to 25 h 28 min (Supplementary Materials File F1). Table 4 has been converted into a histogram for ease of interpretation in Supplementary Materials Figure S4A.

Interestingly, proteases also influence the amount of time the searches take. Above a critical database size (to be determined but from this experiment anywhere between 515 to 29,057 entries), searches take up to three times longer for rAsp-N-released peptides than for tryptic and chymotryptic peptides (Table 4 and Supplementary Materials Figure S4A). We have averaged all search durations across each of the five databases to produce Supplementary Materials Figure S4B, which shows a marked increase in the duration of the search as a function of the number of entries.

The search engines also perform differently with a clear advantage for SEQUEST over Mascot when the large database Homemade95k is searched. For instance, SEQUEST searched rAsp-N-released peptides three time faster than Mascot (Table 4 and Supplementary Materials Figure S4A).

### 3.3. Comparison of Proteases and Their Proteolytic Efficiencies

In this study, we used three orthogonal digestions with proteases of increasing selectivity levels, chymotrypsin (C), trypsin/Lys-C (TL) and rAsp-N (A). 

The success identification rate follows the order previously observed with the number of MS and MS/MS scans (Table 2). Typically, more accessions are identified when using trypsin/Lys-C, followed by rAsp-N and lastly chymotrypsin (Table 3 and Supplementary Materials Figure S3A). The difference in identification success among proteases becomes also more evident with larger databases. The Venn diagrams in Supplementary Materials Figure S3B further exemplify this with the Homemade95k database, as well as indicating how many of the accessions are unique to each of the proteases or shared among them. For instance, when applying the SEQUEST algorithm, 1108 accessions are identified with TL, 674 with A and 385 with C. Only 265 (17%) accessions are shared among TL and A, 79 (5%) among TL and C and 17 (2%) among A and C. A total of 242 (11%) accessions are common to all proteases. The Venn diagram for Mascot is very similar. Even though some proteases yield longer lists of identities, in particular TL, they all complement each other, as attested by the high number of protease-specific identities (e.g., for SEQUEST 522 TL-specific, 150 A-specific and 47 C-specific protein accessions). This is expected because rAsp-N, trypsin/Lys-C and chymotrypsin are completely orthogonal, target different AA residues and consequently produce unique peptides (Supplementary Materials Table S1). 

Protease complementarity was also observed in our previous study where we tested single, double and triple digestions using orthogonal proteases [2]; taking olivetolic acid cyclase (OAC) as an example, we illustrated that full coverage of its AA sequence could only be reached by combining the sequencing data from all the proteases since none of them individually produced 100% coverage. Similarly, in the present study a wider coverage is achieved upon merging all the sequencing information produced by the A, C and TL proteases (Supplementary Materials Tables S1–S5). From these results and those obtained in our previous multiprotease experiment [2], we conclude that trypsin/Lys-C is the best single digestion method systematically yielding the largest number of identifications regardless of the database used. 

Other studies have applied a multiple protease strategy to increase the proteome depth and sequence coverage. As early as 2002, Choudhary and colleagues demonstrated that 93.9% coverage of a recombinant tissue plasminogen activator could be achieved by the combination of trypsin, Lys-C and Asp-N, covering respectively 88.2%, 62.8% and 34.9% of the 527 AA sequence [11]. Whilst covering the least, Asp-N proved essential as it spanned regions of the recombinant protein that were not explored by either trypsin or Lys-C. A similar observation was made on bovine serum albumin [48]. Swaney and colleagues compared trypsin to highly selective proteases, namely ArgC, AspN, GluC and LysC, and observed that while trypsin yielded the greatest number of unique identifications, the alternative proteases identified different proteins thus augmenting the proteome depth [49]. Asp-N ranked second with respect to the number of unique peptides identified, albeit achieving a lesser sequence coverage. 

We mentioned above that perhaps applying similar protease:proteins ratios during the digestion step might lead to comparable success rates among the different proteases. We also need to factor in protease proteolytic efficiencies or how effectively proteases find their target AA residue and cleave their substrate. This is assessed by the number of missed cleavage sites. The manufacturer Promega (https://www.promega.com.au) ranks proteolytic efficiencies as follows: TL > A > C (Figure 1). The website stipulates that trypsin/Lys-C yields less than 10% missed cleavages of R and K residues, thus realizing more than 90% efficiency; rAsp-N achieves 85% digestion efficiency (no missed cleavage) of D residues after 1h. Under our conditions (1M Guanidine-HCl), chymotrypsin loses 20% cleavage efficiency (https://www.promega.com.au) of Y, F and W residues. We must also consider variations in fragmentation efficiencies of the peptides as both trypsin/Lys-C and chymotrypsin leave a proton on the peptide C-terminus, whereas Asp-N leaves it on the N-terminus of the released peptides. 

The SEQUEST search program allows for up to twelve miscleavages whereas Mascot only allows for up to nine miscleavages. We have previously discussed the benefits of setting a number of miscleavages greater than two [2], particularly in the context of middle-down proteomics. Table 5 presents the distribution of missed cleavage sites observed in our experimental data.

The majority (60–89%) of the peptides matched in this study do not contain any miscleavage. However, a significant proportion (11–40%) does include missed cleavage sites, indicating that our digestions are incomplete. This is further confirmed by subtracting the number of matched peptides with a missed cleavage site (miscleavage > 0) from the number of matched peptides without missed cleavage (miscleavage = 0) to compute the excess of limit-digested peptides (ELDP) [50]. If the proteolysis was total, the ELPD values indicated in Table 5 would be much smaller.

The fact that the digestion is incomplete is not an issue in our study. It just warrants allowing for more miscleavages in the search parameters, which will result in longer search times, as was discussed above. However, this is also advantageous in an MDP context where more missed cleavage sites create longer peptides and ultimately greater sequence coverage, as was demonstrated in our previous study [2] and confirmed in this new study. The peptide sizes (i.e., masses) are reported in Table 6.

Identified peptide masses range from 604.3 D (Homemade95k) to 7600.9 D (SP21) and they average 2032.5 D with a huge standard deviation (SD, Table 6A), indicating that the size of many peptides falls outside the average mass. There is a trend that the larger the database, the smaller the identified peptides.

If we take a closer look at the protease level, rAsp-N produces the longest peptides, averaging from 2.0 to 2.5 (+/− 0.9–1.2) kD (Table 6B). This is expected because rAsp-N is highly selective and targets only the N-terminus of D residues. The largest peptide originates from the action of rAsp-N on CBCAS (WO/2015/196275Al), weighs 7.6 kD, hosts only one miscleavage and matches the following AA sequence: DLFWAIRGGGGENFGIIAACKIKLWVPSKATIFSVKKNMEIHGLVKLFNKWQNIAYKYDK. 

Proteases that are less selective and target multiple sites such as trypsin and chymotrypsin produce shorter peptides averaging 1.9 kD. The longest peptides arising from the action of C or TL present more missed cleavages. For instance, the chymotryptic peptide EILSGKSRGAAAATESLTDSSAEFGETSSSISSSEISTEDVKVKGSSSPPHLGWPIRRADVRKSF from the *C. sativa* rop guanine nucleotide exchange factor 5-like protein (XP_030490016.1) weighs 6954.3 D and contains 5 miscleavages. In another example, the tryptic peptide VSRLDLKKLRFGAANRYGFRVGLGKTHLSANFSDEVASWKKFRNQR from the *C. sativa* uncharacterized protein LOC115699895 (XP_030483299.1) weighs 5539.9 D and carries 10 miscleavages. 

In our previous shotgun study, we evidenced the positive relationship between the number of miscleavages and the size of the peptides [2]. We discussed how advantageous this feature is in a middle-down proteomics context and recommended applying a number of missed cleavages greater than two, as is usually the case, during the database search stage. This is also confirmed in the present work. Swaney and colleagues applied up to three missed cleavages and reported increased length of Asp-N-released peptides relative to that of tryptic peptides [49]. Giansanti and colleagues observed 0–2 miscleavages for trypsin and Lys-C and 0-4 miscleavages for Asp-N and chymotrypsin [48]. Cristobal and colleagues reported Asp-N-released peptides bearing more than four miscleavages and a greater median size than tryptic peptides [51].

### 3.4. Comparison of the Search Algorithms

A plethora of search engines are available to the proteomics community to turn tandem mass spectra of peptides into AA sequences [22,25,27,28,29]. All of these algorithms rely upon the same fundamental elements: read protein sequence databases, emulate enzymatic cleavage to peptides, extrapolate PTMs, apply a tolerance of observed precursor and fragment masses, predict fragment ions for each peptide sequence, and compare observed and expected fragments [29]. In our past shotgun proteomics studies [1,2], we used SEQUEST, which was designed for instruments manufactured by Thermo Scientific such as the Elite LTQ-orbitrap mass analyser employed here. In this study we compare two of the most commonly used search algorithms, SEQUEST and Mascot. 

The SEQUEST program was created in 1994 to correlate tandem mass spectra of digested protein mixtures from a yeast cell lysate with AA sequences hosted in a database [23]. Amino acid sequences are converted into a fragmentation pattern used to match fragment ions in a MS/MS spectrum. The number of peaks the sequence shares with the experimental spectrum are counted to generate the SEQUEST preliminary score or Sp [23]. Two key calculations assess whether a peptide sequence is a confident match for a fragmentation spectrum: 1) XCorr, a statistical calculation of the correlation of the theoretical and experimental spectra and 2) ΔCN, the difference between the top peptide spectrum match (PSM) and the second best PSM [8]. SEQUEST was exclusively licensed to Thermo Scientific instruments and incorporated into Proteome Discoverer 1.4 package [29]. Since its inception in 1994, SEQUEST has undergone a series of improvements [29], including the addition of dynamic modifications [52] and the ability to interrogate nucleotide databases through six-frame translation [53]. 

Developed in 1999, the Mascot program incorporates a probability-based scoring which allows discrimination against false positives, can be compared with other probabilities such as sequence homology and can be optimized by iteration [24]. To maximise search speed and reduced data, FASTA format sequence databases are compressed, and multiple spectra originating from the same precursor are summed together. Tandem MS data are converted to peak lists of centroided mass values associated with intensity values. The match significance depends on the size of the database. Fixed and variable (so called dynamic in SEQUEST) modifications can also be included [24]. Several common causes of failure to find a peptide match are considered in the Mascot program: (a) enzyme nonspecificity, (b) incorrect determination of precursor charge, (c) underestimated mass measurement error, (d) unsuspected chemical and post-translational modifications and (e) peptide sequence not in the database [43]. 

A pubmed survey (https://www.ncbi.nlm.nih.gov/pubmed/) with the following key words “proteom* AND mascot” or “proteom* AND sequest” indicates that even though SEQUEST predates Mascot by five years, more proteomics publications contain the term Mascot (751) than the term SEQUEST (346). This can probably be explained by the fact that the SEQUEST search engine is only distributed with a Thermo Scientific instrument whereas Mascot is a stand-alone license that can be purchased independently of the instrument used. The distribution of publications per year is available in Supplementary Materials Table S6.

The number of accessions identified varies slightly depending on which search algorithm is employed, with SEQUEST always yielding more identities (Table 3 and Supplementary Materials Figure S3A). For instance, when Uniprot515 is searched, SEQUEST yields an average of 68 (+/−3) accessions, whereas Mascot produces 41 (+/−5) accessions. When a much larger database like Homemade95k is interrogated, the gain becomes more evident with 1117 (+/−118) SEQUEST-related accessions and 772 (+/−211) Mascot-related accessions. If time is a constraint and very large databases are interrogated, then SEQUEST is to be favoured, with search durations up to three times faster than when Mascot is used, as demonstrated for the largest database, Homemade95k, in Table 4 and illustrated in Supplementary Materials Figure S4A. 

When all the algorithm-specific matches are considered for the four databases where both SEQUEST and Mascot were applied and the results represented as Venn diagrams, it paints a slightly different picture (Supplementary Materials Figure S3C). With the exploration of the SPGP40k database, Mascot identifies 735 (out of 819 IDs, 90%) accessions across all nine samples, thus slightly outperforming SEQUEST, which yields 710 (87%) identifications; 626 (76%) are common between the two algorithms. Overall, irrespective of the database, 61% to 78% of the matches are shared among both algorithms (Supplementary Materials Figure S3C). Some accessions are unique to SEQUEST or to Mascot, thus boosting the number of proteins identified when both programs are taken into account. Therefore, if utilizing several search algorithms is a possibility, prospective researchers should consider it.

To our knowledge, four studies have compared Mascot and SEQUEST search engines on diverse samples [51,54,55,56], however none originating from plants. Shen and colleagues reported that the number of peptides identified using Mascot was only 40–60% of that obtained using SEQUEST, attributed to numerous Mascot-related false negative identifications. Mascot rejected many peptides whose masses fell within the set tolerance and matched unique sequence tags composed of more than seven residues. The authors conclude that Mascot operates better for well-resolved, small and doubly charged peptides [55]. Tu and colleagues also report wide differences between both search engines; however these discrepancies could be leveled out using a post-processing program such as Percolator [56]. Cristobal et al. indicate that Mascot performs better than SEQUEST on deconvoluted MS/MS data because the latter rewards data-rich spectra such as those exhibited by large fragments displaying a wide charge envelop [51]. Very recently, Agten and colleagues debate that resorting to multiple algorithms to search MS/MS data actually hides the information on complementarity and agreement among the search engines at the level of spectrum identification [54]. They stipulate that the percentage sequence agreement on peptide identification at the spectrum level can assess the rate of agreement between the search engines better than a Venn diagram of matched peptides or identified proteins. The combination of both sequence annotation and sequence confidence is achieved using Mondrian-like plots and shows that Mascot matches more tandem spectra than SEQUEST [54]. 

Other studies have utilized additional search engines on top of SEQUEST and Mascot, including Spectrum Mill, X!Tandem, PeptideProphet and Sonar [57], X!Tandem and OMSSA [58], MaxQuant [59], InsPeCT, OMSSA, x!Tandem and MyriMatch [28], Andromeda and SimSpectraST [60], and MaxQuant and Andromeda [60]. The more algorithms, the less the overlap across the identification results, as clearly illustrated with Venn diagrams or scatter plots. This begs the following question: do we consider only the common hits, or do we accept all matches regardless of their origin? We argue that all those search engines have been well designed and validated, and therefore, all peptide hits should be considered. Indeed, multiplying search algorithms has led to improved overall identification numbers and confidence [8,22].

### 3.5. Sequence Coverage and Post-Translational Modifications (PTMs)

Every shotgun proteomics experiment strives at producing the longest list of protein identifications with the broadest sequence coverage possible in order to distinguish between the various isoforms and detect PTMs. 

**Figure 3:**
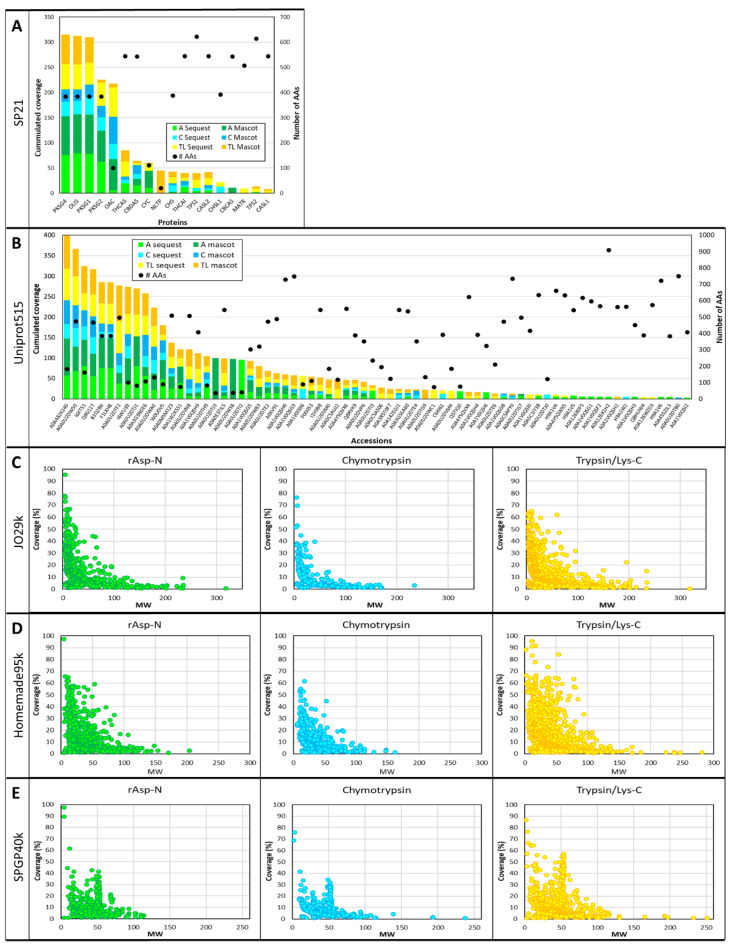
Comparison of the protein coverage results obtained using the five databases. (**A**) Histogram of cumulated sequence coverage for the 18 proteins identified using SP21 database. The secondary Y-axis represents the number of AAs. (**B**) Histogram of cumulated sequence coverage for the 72 accessions identified using Uniprot515 database. The secondary Y-axis represents the number of AAs. (**C**) Scatterplot of the coverage of the 1343 accessions identified using JO29k database plotted against their MWs (kD) for each digestion. (**D**) Scatterplot of the coverage of the 1442 accessions identified using Homemade95k database plotted against their MWs (kD) for each digestion. (**E**) Scatterplot of the coverage of the 819 accessions identified using SPGP40k database plotted against their MWs (kD) for each digestion.

The sequence coverage of all the proteins identified using the five different databases and listed in Supplementary Materials Tables S1–S5 have been turned into histograms and scatterplots factoring in sequence length using the number of AAs or the MWs of the proteins identified in this study (Figure 3). 

When small databases such as SP21 and Uniprot515 are interrogated, there is a trend showing that short proteins achieve greater sequence coverage (Figure 3A,B), albeit with many exceptions. For instance, with SP21, four proteins composed of 385 AAs, 3,5,7-trioxododecanoyl-CoA synthase (OLIS) and polyketide synthases 1, 2 and 4 (PKSG1, PKSG 2 and PKSG 4) are well covered (up to 79% with rAsp-N), whereas other proteins of similar size, such as naringenin-chalcone synthase (CHS) and chalcone synthase-like protein 1 (CHSL1), only reach a maximum of 39% and 27% coverage, respectively (Figure 3A). Other exceptions are olivetolic acid cyclase (OAC) and cytochrome c (CYC), composed of 101 and 111 AAs, respectively; while small, these proteins’ sequences are not completely covered. Similar observations can be made with Uniprot515; generally speaking, small proteins are better covered, but some exceptions are found (Figure 3B). When large databases like JO29k, Homemade95k and SPGP40k are explored, the trend described above becomes very clear and scatterplots confirm the negative relationship between protein MWs and sequence coverage irrespective of the protease used, as can be seen in Figure 3C–E.

The *C. sativa* proteins annotated in the UniProt Knowledge Base are known to carry modifications, and we have experimentally validated some of them using a top-down proteomics strategy [3]. Consequently, in this study we have included the following dynamic PTMs to the search method: N-term acetylation, acetylation and methylation of K residues, oxidation of M residues, phosphorylation of S, T and Y residues and the attachment of N-acetyl-D- glucosamine (NAG) glycogroups to N residues. Examples of *C. sativa* proteins bearing NAG glycosylations are CBDAS (A6P6V9) and THCAS (Q8GTB6) [61,62,63]. Furthermore, following the DTT reduction and iodoacetamide alkylation of proteins during sample preparation, cysteine residues involved in disulfide bonds are expected to be reduced and carbamidomethylated. The number of fixed and dynamic PTMs discovered in this experiment are reported in Table 7.

Depending on the database employed, between 25 and 44% of the identified peptides harbor one or several modifications. The number of PTMs varies from 277 (SP21 and Uniprot515) to 1272 (Homemade95k), again exhibiting a positive relationship with the size of the database. Most PTMs are carbamidomethylations (602/1272, i.e., 47% of all PTMs in Homemade95k, Table 7). This is expected as many proteins comprise disulfide bridges in their secondary structures and as such hold a pivotal role in their folding, stability and activity [64]. 

The proportions of dynamic PTMs fluctuate in a database-dependent fashion. For example, the second largest category of PTMs is phosphorylation for Uniprot515 (57/277, 21%) and Homemade95k (201/1272, 16%) databases, but it is methylation for JO29k (114/833, 14%) and SPGP40k (158/667, 24%) databases (Table 7). Acetylations, whether they are located at the N-terminus of the protein or not, are well represented, particularly with SP21 (47 + 21 = 68/277, 25%), Homemade95k (91 + 132 = 223/1272, 18%) and SPGP40k (44 + 71 = 115/667, 17%). Oxidation only affects a small proportion of peptides (5–13%), suggesting that no artefactual oxidation was introduced during the sample preparation steps. 

In this study, several peptides decorated with NAG (also called GlcNAc) are detected (1–4%). With SP21, they are witnessed on polyketide synthase 2 (PKSG2), cannabichromenic acid synthase (CBCAS), cannabidiolic acid synthase (CBDAS), cannabidiolic acid synthase-like 2 (CBDAS3), tetrahydrocannabinolic acid synthase (THCAS) and inactive tetrahydrocannabinolic acid (THCAI) (Supplementary Materials Table S1). Using Uniprot515, they are additionally found on ocimene synthase (A0A5C1IY38), hedycaryol synthase (A0A4Y5QVX6) and mevalonate kinase (A0A1V0QSI0). Such NAG N-linked glycosylation sites have been reported for CBDAS (https://www.uniprot.org/uniprot/A6P6V9) [62], CBDAS3 (https://www.uniprot.org/uniprot/A6P6W1), THCAS (https://www.uniprot.org/uniprot/Q8GTB6) [61,65], THCAI (https://www.uniprot.org/uniprot/Q33DQ2) and CBCAS (patent WO/2015/196275 Al [41]). Most of these synthases are involved in highly specific secondary metabolisms, the phytocannabinoid, terpenoid and mevalonate pathways. 

PTMs of cannabis proteins have also been reported by Jenkins and Osburn [39]; oxidation of methionine residues was the most common modification, followed by acetylation of lysine residues and phosphorylation of serine and threonine residues. Interestingly, the authors indicate that the acetylated proteins were unique to mature flowers and absent in leaves and stems from the male plants. Protein PTMs represent a major level of cellular regulation, acting either swiftly and reversibly, such as phosphorylation, or slowly and irreversibly, such as certain forms of glycosylation. Whilst gene expression merely regulates protein abundance, PTMs control their three-dimensional structures, thus revealing or concealing active sites and interfaces for protein–protein interaction, which in turn modulates the protein subcellular localization, stability and activity. Acting as molecular switches of proteins, PTMs may initiate and inhibit the interaction of proteins with DNA, cofactors and lipids, as well as with other proteins [66]. The human proteome is the best proteome characterized so far and MS has enabled the discovery of most of the PTMs known today. A catalog of 81,721 unique phosphorylated peptides belonging to 11,025 proteins substrates of kinases, 29,031 unique ubiquitinylated peptides corresponding to 5769 proteins substrates of ubiquitin ligases, 16,693 acetylated peptides from 7098 proteins that are substrates of acetylases and 7977 proteins and carboxy-terminal peptides for 6778 proteins confirming a large number of translation start and stop sites have been established [67]. As evidenced in this study and previous works [1,2,39], *C. sativa* hosts numerous PTMs with many more to be discovered as the number of proteomics experiments on *C. sativa* gain momentum, leveraged by a relaxation in the legislation. Neither genomics nor transcriptomics analysis can identify PTMs, only protein analyses can deliver such valuable information. Experimentally detecting PTMs using MS is a first step; functionally characterizing them is another critical step that is needed in order to shed more light onto the biology of this unique plant. 

### 3.6. Database Specificity and Gene Ontology (GO)

Four databases contain exclusive sequences from *C. sativa*, whereas SPGP40k includes all the sequences from SwissProt limited to the *viridiplantae* (i.e., green plants) taxonomy. The 819 identifications obtained in this study using the green plant taxonomy emanate from 175 different plant species. The histogram in Supplementary Materials Figure S5 displays 32 species represented by more than 4 accessions.

Most identities (267/819, 33%) come from *Arabidopisis thaliana*, which is the model plant species and therefore the most studied, sequenced and best annotated organism. Then, *Oryza sativa*, the cereal model, ranks second with 67 (8%) accessions. Only eight (1%) accessions originate from *C. sativa*, which ranks 11th and is equally placed with *Cucumis sativus* and *Gossypium hirsutum* (Supplementary Materials Table S5). The SPGP40k database comprises 19 *C. sativa* (CANSA) entries, which corresponds to only 0.05% of the number of total entries. All the 19 CANSA entries are also included into the SP21 database, which yields 18 accessions when searched in this study (Supplementary Materials Table S1). Therefore, it is not clear why only 8 (out of 21, 42%) CANSA accessions are found when SPGP40k is searched; it is as though they underwent a database dilution. Because decoy searches were performed and PSM validated, we do not expect false positives to occur in our analyses. Sequences from non-model species such as *C. sativa* are greatly underrepresented in the most reputable protein reference database, UniProt, even though this species’ genome has been sequenced and annotated in NCBI. It would be useful for the proteomics community to have these sequences and all their known annotations (e.g., GO terms, PTMs, signal peptide, etc.) available from the UniProt Knowledge Base. Unsequenced organisms primarily face the challenge of bioinformatic data analysis, particularly when no close relatives have been sequenced [68]. The proteome of another non-model species, *Artemisia annua*, was also mined using various databases originating from RNA sequencing or retrieved from NCBInr and UniProt repositories. The searched databases were limited to only *A. annua* species or included the whole *viridiplantae* taxonomy; accordingly, the number of entries ranged from 118 to more than 1 million sequences [69]. Large specific databases led to the identification of almost 700 accessions, whereas *viridiplantae* databases listed about half that number. Similar conclusions were drawn more recently on cacao, also a non-model species [70]. The authors report that the largest number of identities (906) were obtained using a database made of the *Theobroma cacao* genomic sequences translated into six reading frames and containing 59,577 entries. The *T. cacao* UniProt/Trembl and NCBI databases yielded 897 and 870 protein identifications, respectively. NCBInr *viridiplantae*, the largest database searched in this study in excess of 3 million entries, identified 759 proteins. Both these works thus demonstrate that database specificity rather than exhaustivity is a key factor to consider for proteomics analyses; we also report this on *C. sativa*. When studying non-model plant species for which no genomic sequencing data is available, searching the *viridiplantae* database and its sub-taxonomies has proven invaluable to explore their proteomes, as was evidenced in pomegranate [71], quinoa [72], *Pinus occidentalis* [73] and cumin [74]. 

Using the UniProtKB Retrieve/ID mapping online tool (https://www.uniprot.org/uploadlists/) and the Uniprot accessions identified using Uniprot515 and SPGP40k databases, we performed a GO classification; this is not feasible with accession numbers from MPGR and NCBI. The longer the list of annotations, the more exhaustive the insight into the plant biology, as displayed in Supplementary Materials Figure S6 based on the Biological Processes classification.

As more proteins are identified when a large database such as SPGP40k is used (Supplementary Materials Figure S6B) relative to a small database such as Uniprot515 (Supplementary Materials Figure S6A), more biological processes are listed. In the case of medicinal cannabis, 581 metabolic processes appear including nitrogen compounds (340), primary (452), small molecules (224) and organic substances (508) metabolisms. Yet, only 7 results are assigned to the secondary metabolism. *C. sativa* manufactures a plethora of compounds found nowhere else, the best known being the phytocannabinoids [30,31,32,33,34]. This is not reflected in the classification depicted in Supplementary Materials Figure S6B because no other species resembles cannabis, which is truly unique. This *viridiplantae* database gap in SwissProt and UniProt must be urgently filled.

## 4. Conclusions

In this BUP experiment, three orthogonal proteases of various selectivity were applied to mature buds of *C. sativa* and analysed using nLC-MS/MS. Five databases of various sizes and specificity and two search engines were used to explore the spectral data. Statistical analyses, PCA and Venn diagrams in particular, highlight the complementarity of the proteases. A portion of the peptides identified in this study are shared across two or more proteases but many of them are unique to a digestion. Overall, not only more accessions are identified but also with greater sequence coverage and numerous PTMs discovered. Searching five databases of increasing sizes, from a minimalist database representing only a pathway (SP21) to a redundant database containing more than the expected number of *C. sativa* genes (Homemade95k), revealed a positive relationship between the number of entries in the database and the number of identities. A negative consequence is that the duration of the search increases accordingly, spanning from mere minutes with small databases to over a day with large ones. The two search engines SEQUEST and Mascot performed adequately, with a slight advantage given to SEQUEST, which yielded slightly more identifications in a shorter amount of time. Most accessions were shared among the two algorithms but a significant proportion of them were unique to one of them. Therefore, like for the proteases, multiplying the search engine is beneficial as it yields more identities and ultimately provides a better biological insight. To the prospective scientist devising a shotgun proteomics strategy to explore the proteome of their samples, we recommend performing multiple digestions, to search databases that aptly represent the gene diversity of their species of interest and to utilise multiple search engines if possible.

## Figures and Tables

**Table 1:** Description of the five FASTA databases used in this study.

DB Name	Source	Number of Entries	Annotation	Date	Algorithm	Taxonomy
SP21	https://www.uniprot.org/uniprot/?query=taxonomy:%22Rosales%20[3744]%22%20cannabis%20organism:sativa&fil=reviewed%3Ayes https://patentscope.wipo.int/search/en/detail.jsf?docId=WO2015196275 https://patentscope.wipo.int/search/en/detail.jsf?docId=WO2011017798&_cid=P11-K8DWCD-64087-1	19 from SwissProt + CBCAS (patent WO2015/196275) + GOT (patent WO2011/017798) = 21	Yes	Feb 2020	SEQUEST Mascot	C. sativa
Uniprot515	https://www.uniprot.org/uniprot/?query=taxonomy%3A%22Rosales+%5B3744%5D%22+cannabis+organism%3Asativa https://patentscope.wipo.int/search/en/detail.jsf?docId=WO2015196275 https://patentscope.wipo.int/search/en/detail.jsf?docId=WO2011017798&_cid=P11-K8DWCD-64087-1	513 from UniProt + 2 patents = 515	Yes	Feb 2020	SEQUEST Mascot	C. sativa
JO29k	https://www.cannabisdraftmap.org/	29,057	Yes	Dec 2019	SEQUEST ^1^	C. sativa
Homemade95k	https://www.uniprot.org/uniprot/?query=taxonomy%3A%22Rosales+%5B3744%5D%22+cannabis+organism%3Asativa https://www.ncbi.nlm.nih.gov/protein (cannabis sativa) AND "Cannabis sativa"[porgn:__txid3483] http://medicinalplantgenomics.msu.edu/pub/data/MPGR/Cannabis_sativa/	Uniprot515 + 37,143 + 57,411 = 95,069	Yes Yes No	Feb 2020	SEQUEST Mascot	C. sativa
SPGP40k	https://www.uniprot.org/uniprot/?query=reviewed:yes%20taxonomy:33090	39,800	Yes	Feb 2020	SEQUEST Mascot	Green plants

^1^ JO29k FASTA file could not be parsed in Mascot due to duplicate rows. SEQUEST could handle the duplicates.

**Table 2:** Number of MS and MS/MS scans and clusters per sample.

Sample	MS Scans	MS/MS Scans	MS Clusters
bud1_A	12,582	10,990	91,784
bud2_A	11,820	10,174	85,566
bud3_A	11,686	10,079	85,388
bud1_C	11,345	9532	89,030
bud2_C	10,391	8458	82,091
bud3_C	11,562	9597	83,440
bud1_TL	13,423	11,828	91,320
bud2_TL	12,858	11,242	87,335
bud3_TL	12,330	10,665	84,845
mean A	12,029	10,414	87,579
SD A	483	501	3642
CV A	4	5	4
mean C	11,099	9196	84,854
SD C	623	640	3679
CV C	6	7	4
mean TL	12,870	11,245	87,833
SD TL	547	582	3266
CV TL	4	5	4

**Table 3:** Number of identities for each sample across the five databases and the two algorithms.

Database	# Proteins in Database	Sample	# Proteins with SEQUEST	# Proteins with Mascot	% Proteins with SEQUEST	% Proteins with Mascot
SP21	21	bud1_A	15	9	71.4	42.9
SP21	21	bud2_A	15	9	71.4	42.9
SP21	21	bud3_A	15	9	71.4	42.9
SP21	21	bud1_C	15	12	71.4	57.1
SP21	21	bud2_C	15	12	71.4	57.1
SP21	21	bud3_C	15	11	71.4	52.4
SP21	21	bud1_TL	16	15	76.2	71.4
SP21	21	bud2_TL	15	14	71.4	66.7
SP21	21	bud3_TL	16	16	76.2	76.2
Uniprot515	515	bud1_A	65	40	12.6	7.8
Uniprot515	515	bud2_A	63	35	12.2	6.8
Uniprot515	515	bud3_A	67	36	13.0	7.0
Uniprot515	515	bud1_C	67	46	13.0	8.9
Uniprot515	515	bud2_C	70	39	13.6	7.6
Uniprot515	515	bud3_C	70	38	13.6	7.4
Uniprot515	515	bud1_TL	70	48	13.6	9.3
Uniprot515	515	bud2_TL	69	39	13.4	7.6
Uniprot515	515	bud3_TL	69	48	13.4	9.3
JO29k	29,057	bud1_A	1071	n.a.	3.7	n.a.
JO29k	29,057	bud2_A	1037	n.a.	3.6	n.a.
JO29k	29,057	bud3_A	1034	n.a.	3.6	n.a.
JO29k	29,057	bud1_C	748	n.a.	2.6	n.a.
JO29k	29,057	bud2_C	766	n.a.	2.6	n.a.
JO29k	29,057	bud3_C	807	n.a.	2.8	n.a.
JO29k	29,057	bud1_TL	1244	n.a.	4.3	n.a.
JO29k	29,057	bud2_TL	1162	n.a.	4.0	n.a.
JO29k	29,057	bud3_TL	1188	n.a.	4.1	n.a.
Homenade95k	95,069	bud1_A	1130	792	1.2	0.8
Homenade95k	95,069	bud2_A	1115	741	1.2	0.8
Homenade95k	95,069	bud3_A	1085	699	1.1	0.7
Homenade95k	95,069	bud1_C	981	552	1.0	0.6
Homenade95k	95,069	bud2_C	988	555	1.0	0.6
Homenade95k	95,069	bud3_C	1002	549	1.1	0.6
Homenade95k	95,069	bud1_TL	1322	1126	1.4	1.2
Homenade95k	95,069	bud2_TL	1192	922	1.3	1.0
Homenade95k	95,069	bud3_TL	1237	1009	1.3	1.1
SPGP40k	39,800	bud1_A	627	439	1.6	1.1
SPGP40k	39,800	bud2_A	620	415	1.6	1.0
SPGP40k	39,800	bud3_A	605	394	1.5	1.0
SPGP40k	39,800	bud1_C	604	443	1.5	1.1
SPGP40k	39,800	bud2_C	605	395	1.5	1.0
SPGP40k	39,800	bud3_C	621	416	1.6	1.0
SPGP40k	39,800	bud1_TL	756	688	1.9	1.7
SPGP40k	39,800	bud2_TL	706	562	1.8	1.4
SPGP40k	39,800	bud3_TL	730	624	1.8	1.6

**Table 4:** Search times across the five databases and two algorithms for each sample.

Database	Sample	Total Search Duration ^1^	SEQUEST/Decoy ^2^ Search Duration	Mascot/Decoy ^2^ Search Duration
SP21	bud1_A	11 min 0 s	2 min 0 s	6 min 43 s
SP21	bud2_A	10 min 0 s	1 min 30 s	6 min 44 s
SP21	bud3_A	10 min 0 s	1 min 31 s	6 min 25 s
SP21	bud1_C	10 min 0 s	2 min 35 s	4 min 52 s
SP21	bud2_C	8 min 0 s	1 min 54 s	4 min 4 s
SP21	bud3_C	10 min 0 s	2 min 21 s	5 min 12 s
SP21	bud1_T	12 min 0 s	2 min 28 s	6 min 42 s
SP21	bud2_T	11 min 0 s	2 min 18 s	6 min 28 s
SP21	bud3_T	11 min 0 s	2 min 12 s	6 min 1 s
Uniprot515	bud1_A	20 min 0 s	5 min 30 s	10 min 12 s
Uniprot515	bud2_A	19 min 0 s	5 min 10 s	10 min 53 s
Uniprot515	bud3_A	21 min 0 s	5 min 15 s	11 min 42 s
Uniprot515	bud1_C	18 min 0 s	8 min 28 s	5 min 12 s
Uniprot515	bud2_C	16 min 0 s	7 min 1 s	4 min 22 s
Uniprot515	bud3_C	19 min 0 s	8 min 53 s	5 min 4 s
Uniprot515	bud1_T	26 min 0 s	11 min 33 s	8 min 25 s
Uniprot515	bud2_T	20 min 0 s	8 min 55 s	6 min 4 s
Uniprot515	bud3_T	21 min 0 s	8 min 49 s	7 min 22 s
JO29k	bud1_A	1 h 14 min 0 s	1 h 9 min	n.a.
JO29k	bud2_A	1 h 17 min 0 s	1 h 13 min	n.a.
JO29k	bud3_A	1 h 22 min 0 s	1 h 18 min	n.a.
JO29k	bud1_C	28 min 0 s	24 min 3 s	n.a.
JO29k	bud2_C	19 min 0 s	16 min 14 s	n.a.
JO29k	bud3_C	25 min 0 s	21 min 4 s	n.a.
JO29k	bud1_T	56 min 0 s	51 min 50 s	n.a.
JO29k	bud2_T	45 min 0 s	40 min 29 s	n.a.
JO29k	bud3_T	49 min 0 s	44 min 30 s	n.a.
Homemade95k	bud1_A	19 h 13 min 0 s	4 h 47 min	14 h 17 min
Homemade95k	bud2_A	22 h 16 min 0 s	5 h 14 min	16 h 54 min
Homemade95k	bud3_A	25 h 28 min 0 s	5 h 56 min	19 h 24 min
Homemade95k	bud1_C	8 h 31 min 0 s	2 h 53 min	5 h 31 min
Homemade95k	bud2_C	5 h 21 min 0 s	1 h 31 min	3 h 43 min
Homemade95k	bud3_C	5 h 29 min 0 s	1 h 57 min	3 h 25 min
Homemade95k	bud1_T	9 h 20 min 0 s	2 h 50 min	6 h 22 min
Homemade95k	bud2_T	5 h 29 min 0 s	1 h 49 min s	3 h 30 min
Homemade95k	bud3_T	8 h 10 min 0 s	2 h 19 min s	5 h 43 min
SPGP40k	bud1_A	6 h 48 min 0 s	3 h 33 min	3 h 8 min
SPGP40k	bud2_A	7 h 41 min 0 s	3 h 50 min	3 h 45 min
SPGP40k	bud3_A	8 h 39 min 0 s	4 h 17 min	4 h 15 min
SPGP40k	bud1_C	3 h 35 min 0 s	2 h 3 min	1 h 26 min
SPGP40k	bud2_C	2 h 18 min 0 s	1 h 14 min	59 min 41 s
SPGP40k	bud3_C	2 h 42 min 0 s	1 h 39 min	57 min 18 s
SPGP40k	bud1_T	4 h 22 min 0 s	2 h 27 min	1 h 48 min
SPGP40k	bud2_T	2 h 43 min 0 s	1 h 34 min	1 h 2 min
SPGP40k	bud3_T	3 h 42 min 0 s	1 h 59 min	1 h 36 min

^1^ The total search duration is the time PD 1.4 takes to completely process one LC-MS/MS file as detailed in the workflow supplied in Supplementary Materials Figure S1. Beside database/decoy searches using SEQUEST and Mascot, the workflow includes a spectrum file reading step, a spectrum selector step and a target decoy PSM validator step. ^2^ Decoy searches are performed during the search engine steps using a decoy reversed database; false positives are eliminated during the target decoy PSM validator step. We exemplify this in Supplementary Materials File F1.txt using the Homemade95k database.

**Table 5:** Number of missed cleavages per database.

# Miscleavage	SP21	Uniprot515	JO29k	Homemade95k	SPGP40k
0	116	433	2822	5818	2060
1	33	95	282	1091	403
2	20	51	32	339	140
3	7	16	13	158	60
4	8	9	5	54	28
5	1	1	6	22	7
6	4	3	4	8	5
7	2	3	1	8	4
8	1	0	3	5	1
10	1	0	1	1	1
TOTAL	193	611	3169	7504	2709
TOTAL miscleavage = 0	116	433	2822	5818	2060
TOTAL miscleavage > 0	77	178	347	1686	649
% miscleavage > 0	39.9	29.1	10.9	22.5	24.0
ELPD ^a^	39	255	2475	4132	1411

^a^ ELDP, excess of limit-digested peptides.

**Table 6:** Masses of identified peptides across all five databases (A) and for each protease (B).

A. Peptide Mass	SP21	Uniprot515	JO29k	Homemade95k	SPGP40k
min	626.4	626.4	969.5	604.3	604.3
max	7600.9	6385.2	6724.5	6993.1	6448.6
average	2123.2	2023.2	2173.6	1975.8	1866.0
SD	1099.7	1048.9	791.1	830.3	776.8
**B. Protease**	**Database**	**min Mass**	**max mass**	**average Mass**	**SD Mass**
A	SP21	1006.6	7600.9	2475.2	1166.7
A	Uniprot515	631.3	5994.1	2363.4	1192.1
A	JO29k	969.5	6724.5	2280.9	905.8
A	Homemade95k	653.4	6375.2	2147.2	939.1
A	SPGP40k	653.4	6448.6	2028.9	929.2
C	SP21	774.4	5520.9	1807.1	927.0
C	Uniprot515	704.4	5520.9	1779.1	793.0
C	JO29k	1034.6	6061.9	2108.9	776.2
C	Homemade95k	789.5	6954.3	1901.9	724.2
C	SPGP40k	789.5	5121.4	1832.0	581.4
TL	SP21	626.4	5303.5	2007.0	1058.9
TL	Uniprot515	626.4	6385.2	1926.4	1015.7
TL	JO29k	1055.5	6369.2	2112.1	705.8
TL	Homemade95k	604.3	6369.2	1922.4	789.4
TL	SPGP40k	604.3	6369.2	1795.0	706.0

**Table 7:** Number of post-translational modifications (PTMs) per database.

PTM	SP21	Uniprot515	JO29k	Homemade95k	SPGP40k
Carbamidomethyl (C)	34	94	493	602	226
N-term acetyl (K)	21	16	27	91	44
Acetyl (K)	47	32	47	132	71
Methyl (K)	61	49	114	163	158
NAG (N)	10	5	9	17	7
Oxidation (M)	18	24	43	66	90
Phospho (STY)	86	57	100	201	71
TOTAL PTMs	277	277	833	1272	667
# identified peptides	344	611	3169	7504	2709
# unmodified peptides	192	450	2255	5593	1834
# modified peptides	152	161	914	1911	875
% modified peptides	44.2	26.4	28.8	25.5	32.3

## Supplementary Materials

The following are available online at https://www.mdpi.com/2227-7382/8/2/13/s1, Supplementary Figure S1: Proteome Discoverer 1.4 search parameters, Supplementary Figure S2: Base peak chromatograms (BPCs) of the nine digests, Supplementary Figure S3: Comparison of the number of identifications for each database, search algorithm and protease, Supplementary Figure S4: Comparison of the search times across the databases, algorithms and proteases, Supplementary Figure S5: Distribution of species for which more than four accessions were identified using SPGP40k database, Supplementary Figure S6: Biological Process GO classification for the accessions identified using Uniprot515 (A) or SPGP40k (B) from UniProtKB Retrieve/ID mapping online tool, Supplementary Table S1: SP21 identification results, Supplementary Table S2: Uniprot515 identification results, Supplementary Table S3: JO29k identification results, Supplementary Table S4: Homemade95k identification results, Supplementary Table S5: SPGP40k identification results, Supplementary Table S6: Pubmed publications containing the keywords “proteom* AND sequest” and “proteom* AND mascot”.
