# Supplementary material for: The Power of Three in Cannabis Shotgun Proteomics: Proteases, Databases and Search Engines
Source: Proteomes. 2020 Jun 15;8(2):13. doi: 10.3390/proteomes8020013 (PMC7356525; doi:10.3390/proteomes8020013)
Supplement: Supplementary file 1 [file proteomes-08-00013-s001.zip › proteomes-832887-supplementary/proteomes-832887-supp.-pub/Vincent et al_Proteomes_suppl figures_2020-06-12.pdf]

Vincent D, Savin K, Rochfort S, and Spangenberg G

"The power of three in cannabis shotgun proteomics: proteases, databases and search engines"

Supplementary Figures

Supplementary Figure S1: Proteome Discoverer 1.4 search parameters. The first step “Spectrum Files” uploads the RAW files. The second step “Spectrum selector” applies some filters and specifies the relevant mass spectrometer parameters. The third step is a parallel search using SEQUEST and Mascot algorithm and each of the protease used in this study (see first inset). First the target database (one of the five protein databases indicated in the second inset) is explored, and then the decoy reversed database is searched. The last step is the peptide-spectrum match (PSM) validation comparing the results from both target and decoy databases and eliminating the false positives using a strict false discovery rate (FDR) threshold.

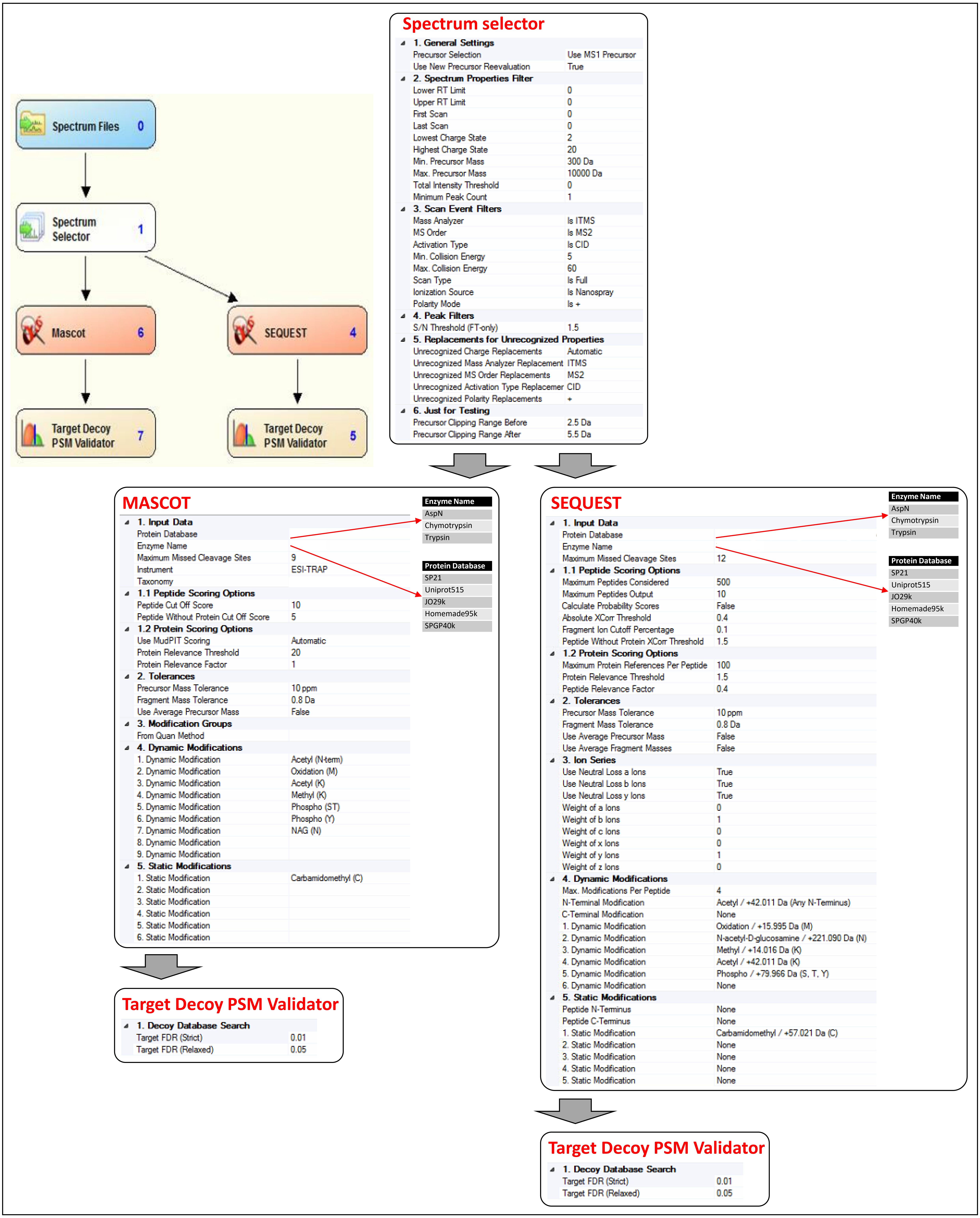

Supplementary Figure S2: Base Peak Chromatograms (BPCs) of the nine digests. Bud 1 to 3 correspond to the three biological replicates.

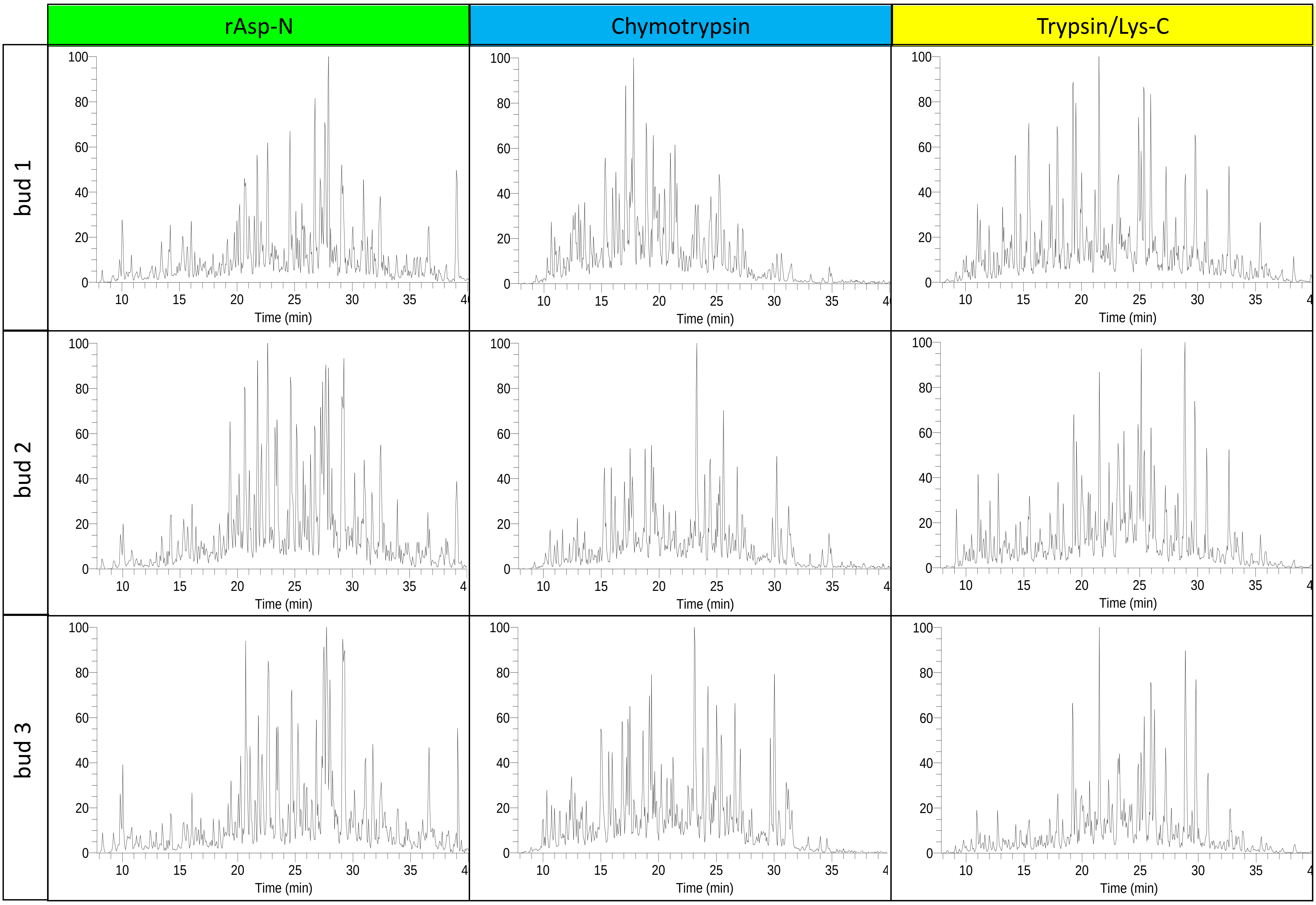

Supplementary Figure S3: Comparison of the number of identifications for each database, search algorithm, and protease. A. Histogram representing the number of accessions averaged per protease for each database and algorithm. The percentages indicated in Table 3 are indicated along the secondary y-axis labelled "Percent". B. Venn diagrams comparing the three digestions for the database Homemade95k and each search algorithm. C. Venn diagrams comparing the two search algorithms for four databases. In brackets are indicated the total number of accessions identified (IDs) for each database.

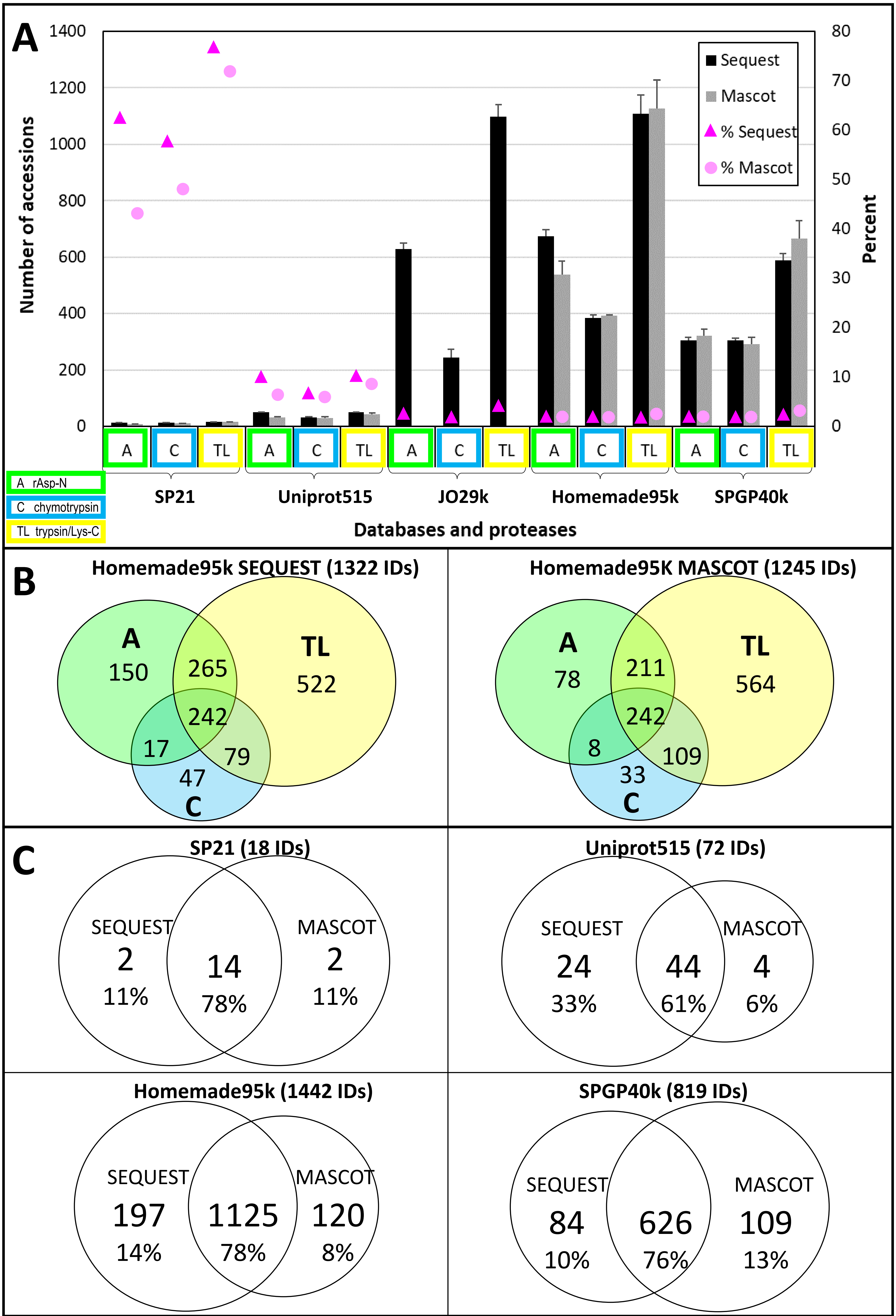

Supplementary Figure S4: Comparison of the search times across the databases, algorithms and proteases. A. Histogram of the times in minutes averaged across the triplicates. Inset displays a smaller time scale for SP21, Uniprot515 and JO29k. B. Scatterplot of the times averaged across the databases.

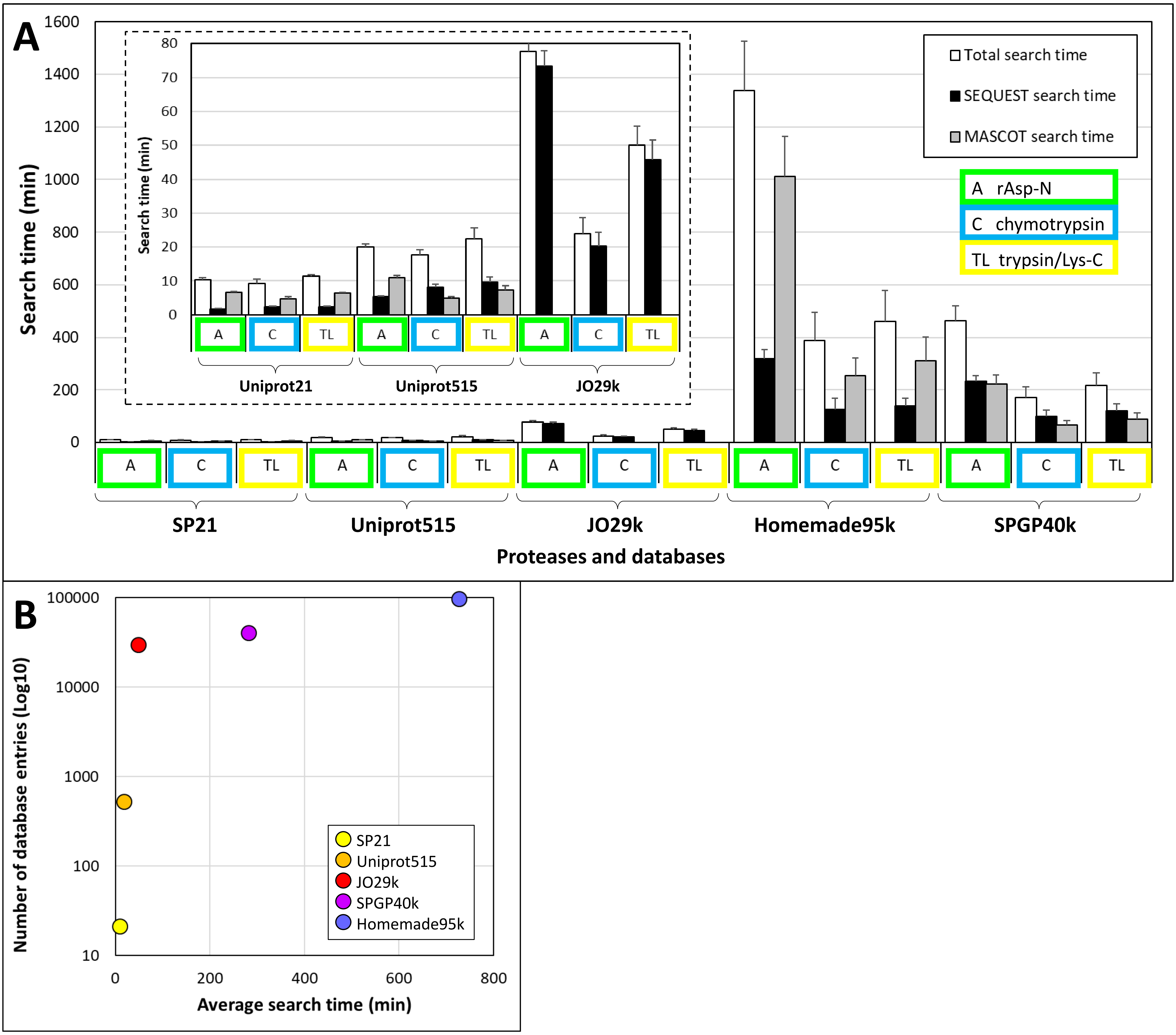

Supplementary Figure S5: Distribution of species for which more than four accessions were identified using SPGP40k database.

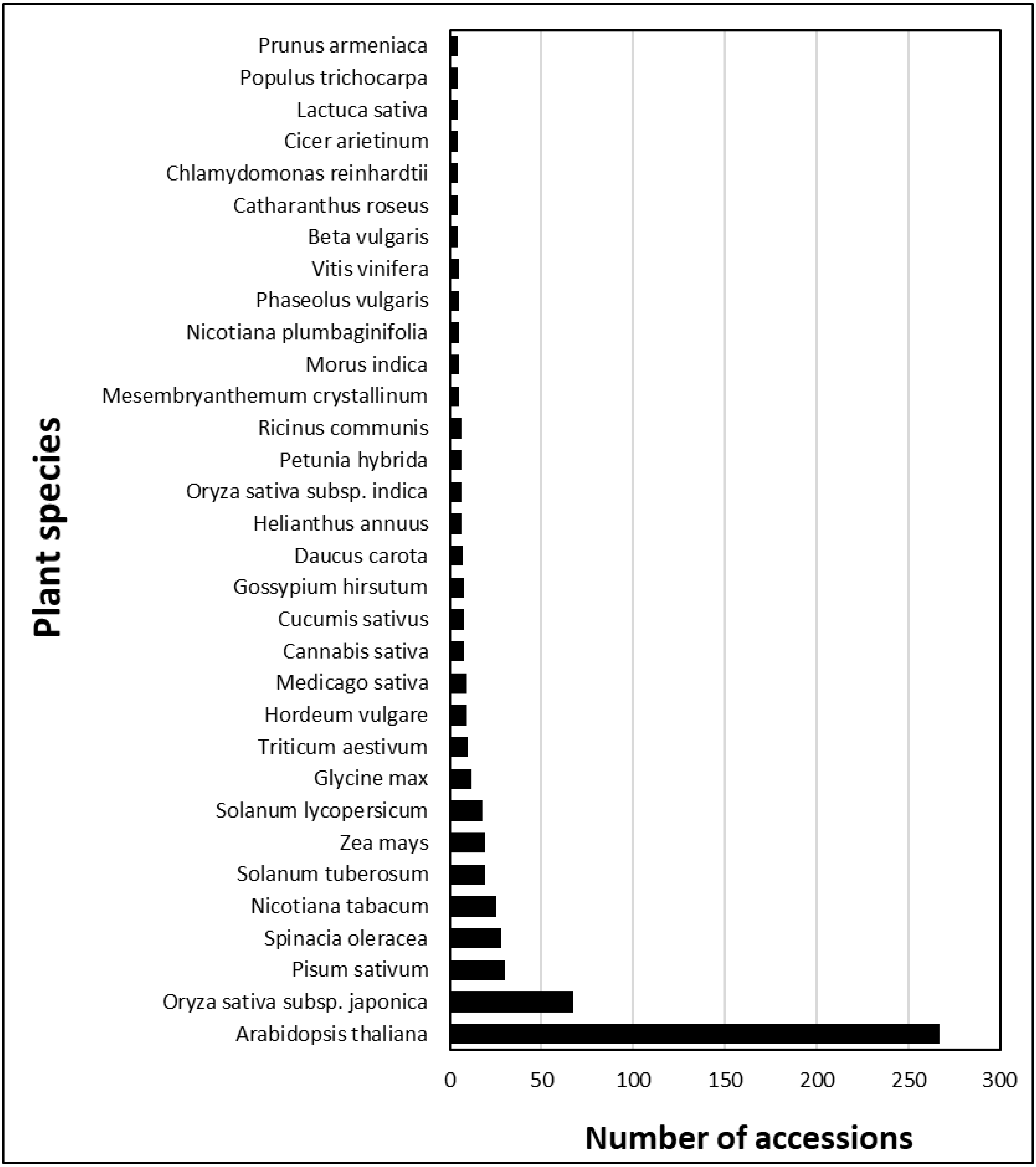

Suppl Figure S6: Gene Ontology (GO) Biological Process classification for the accessions identified using Uniprot515 (A) or SPGP40k (B) from UniProtKB Retrieve/ID mapping online tool.

A. 72 IDs from Uniprot515

- biological_process (50 results)
  - transport (6 results)
  - metabolic process (48 results)
    - nitrogen compound metabolic process (21 results)
    - biosynthetic process (33 results)
    - ATP synthesis coupled proton transport (5 results)
    - secondary metabolic process (2 results)
    - electron transport chain (7 results)
    - cellular metabolic process (45 results)
    - primary metabolic process (34 results)
    - small molecule metabolic process (10 results)
    - organic substance metabolic process (36 results)
  - cellular process (46 results)
  - response to stimulus (2 results)
  - biological regulation (2 results)

B. 819 IDs from SPGP40k

- biological_process (713 results)
  - immune system process (5 results)
  - cell adhesion (1 results)
  - circadian rhythm (5 results)
  - metabolic process (581 results)
    - protein glycosylation (3 results)
    - NADH metabolic process (4 results)
    - NADP metabolic process (7 results)
    - nitrogen compound metabolic process (340 results)
    - catabolic process (128 results)
    - biosynthetic process (330 results)
    - secondary metabolic process (7 results)
      - phenylpropanoid metabolic process (3 results)
      - phenylacetate catabolic process (1 results)
      - glycosinolate metabolic process (2 results)
      - secondary metabolite biosynthetic process (5 results)
      - olivetolic acid biosynthetic process (1 results)
    - methylation (6 results)
    - pigment metabolic process (16 results)
    - hormone metabolic process (3 results)
    - cellular metabolic process (537 results)
    - primary metabolic process (452 results)
    - small molecule metabolic process (224 results)
    - ATP metabolic process (76 results)
    - oxidation-reduction process (41 results)
    - organic substance metabolic process (508 results)
  - cellular process (628 results)
  - pollen tube guidance (1 results)
  - reproductive process (19 results)
  - killing of cells of other organism (1 results)
  - multicellular organismal process (32 results)
  - developmental process (40 results)
  - multicellular organism reproduction (1 results)
  - growth (5 results)
  - response to stimulus (174 results)
  - localization (77 results)
  - multi-organism process (34 results)
  - biological regulation (116 results)
  - cellular component organization or biogenesis (85 results)
  - detoxification (8 results)
